# Effectiveness of chemotherapy after anti‐PD‐1 blockade failure for relapsed and refractory Hodgkin lymphoma

**DOI:** 10.1002/cam4.3262

**Published:** 2020-09-02

**Authors:** Beatrice Casadei, Lisa Argnani, Alice Morigi, Ginevra Lolli, Alessandro Broccoli, Cinzia Pellegrini, Laura Nanni, Vittorio Stefoni, Paolo E. Coppola, Matteo Carella, Michele Cavo, Pier Luigi Zinzani

**Affiliations:** ^1^ Institute of Hematology “L. e A. Seràgnoli” University of Bologna Bologna Italy

**Keywords:** checkpoint inhibitors, chemotherapy, Hodgkin lymphoma, immunotherapy, PD‐1

## Abstract

Programmed death‐1 (PD1) blockade is an efficient and safe therapeutic option in patients with relapsed/refractory (R/R) classical Hodgkin lymphoma (cHL). However, a substantial proportion of patients’ progresses or loses the response to anti‐PD1 treatment. We retrospectively investigated the effectiveness of salvage chemotherapies (CHT) for unsatisfactory response to anti‐PD1, in 25 R/R cHL patients. Twenty‐three patients (92%) were refractory to the last treatment before anti‐PD1. After a median of 14 cycles (range 3‐52), 68% (17/25) of patients had unsatisfactory responses to anti‐PD1 therapy, whereas 6 had a partial response (PR) and 2 patients achieved complete response (CR), with an overall response rate (ORR) of 32%. After a median time of 1.5 months, 15 patients received a single agent treatment and 10 had a multi‐agents regimen, due to the failure of PD1 blockade. The ORR was 60% (8 CR and 7 PR). Seven patients (3 in PR and 4 in CR) underwent a consolidation strategy with stem cell transplantation. Median progression‐free survival (PFS) with salvage treatment was reached at 19.1 months, while median PFS after anti‐PD1 has been reached at 8.2 months. After a median follow‐up of 32.4 months, 6 patients died while 13 are still in CR. The median overall estimated from the start of CHT was not reached. The efficacy of treatment following anti‐PD1 is not yet established, especially in lymphoma patients. To note, in our series, a subset of heavily pre‐treated and chemo‐refractory patients increased response rates to and survival with CHT given after exposure to immune‐checkpoint inhibitors.

## Graphical abstract

A substantial proportion of Hodgkin lymphoma patients´ progresses or loses the response to anti‐PD1 treatment. In our series, a subset of heavily pre‐treated and chemo‐refractory patients increased response rates to and survival with chemotherapy given after exposure to immune‐checkpoint inhibitors.

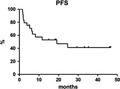

## 1 INTRODUCTION

Despite most patients affected by classical Hodgkin lymphoma (cHL) can be cured with the first line treatment, there is a subset of patients with relapsed/refractory (R/R) disease that still represents an unmet clinical need. Non‐cross resistant salvage chemotherapy followed by autologous stem cell transplantation (ASCT) can save roughly the 50% of R/R patients.^1^, ^2^ According to historical data, patients who failed ASCT have an extremely poor prognosis with a median overall survival (OS) of 2 years.^3^ In this setting, treatment with anti‐CD30 antibody drug‐conjugated brentuximab vedotin (BV) has resulted in high objective response rate (ORR), with 35% of patients obtaining a complete response (CR).^4^ Despite a small subset of CR patients maintains a durable disease control and seems to be cured with BV as single agent, most patients require additional treatment within 1 year.^5^, ^6^ It is already established that HL is able to escape the immune system likely as a result of the amplification of chromosome band 9p24.1, leading to the upregulation of programmed death ligands and JAK/STAT pathway. The use of anti‐Programmed Death‐1 (PD1) monoclonal antibodies (check‐point inhibitors [CPi], namely nivolumab and pembrolizumab) for HL patients who are R/R to ASCT and/or to BV has shown a good balance between efficacy and toxicity, proving to be a good therapeutic option in a subset of such highly pretreated patients. Nevertheless, almost the 70% of patients treated with CPi reached an objective response, but only one third of them obtained a CR and a large part of the responder patients relapses, with a median progression free survival (PFS) of maximum 12 months.^7^, ^8^, ^9^, ^10^, ^11^ To date, what is the optimal treatment after anti‐PD1 failure is still an open question. Retrospective analysis in various tumor types and two multi‐centric series in HL showed a potential improvement in response rate to chemotherapy (CHT) after exposure to CPi, suggesting that this kind of treatment could re‐sensitize the tumor cells to CHT.^12^, ^13^ Here, we report our monocentric experience in heavily pre‐treated and chemo‐refractory HL patients treated with salvage chemotherapy after anti‐PD1 blockade failure, supporting the hypothesis of a new chemo sensitization after CPi.

## 2 METHODS

We retrospectively investigated the effectiveness of salvage therapies for unsatisfactory response to anti‐PD1 therapy in 25 patients with R/R cHL treated with pembrolizumab or nivolumab from March 2015 to December 2018. Regarding salvage approaches, the choice between multi‐agent or single‐agent chemotherapy, immunotherapy or transplantation was made mainly on the basis of the therapies performed pre‐CPi, avoiding treatments already administered when possible. The choice was based on age, performance status and tolerance of previous treatments. In particular, regarding transplantation, the choice was also based on whether the patient had already received an ASCT or not and whether a related donor was available. The patient list was extracted from the electronic database of our Institute. The study was approved by our institutional board and by our Ethical Committee and has been performed in accordance with the ethical standards as laid down in the 1964 Declaration of Helsinki and its later amendments. Patients were consecutively enrolled to avoid selection bias, and all patients provided written informed consent to collect retrospectively their data. We obtained a special permission (for scientific purpose) from our Ethical Committee to collect even data of patients who were deceased or lost to follow‐up. To be enrolled patients must have received at least two cycles of single agent anti‐PD1 and must have unsatisfactory response to CPi (progression disease [PD], partial response [PR] or a CR with a duration less than 3 months). The diagnosis of cHL was established from lymph node biopsies, in accordance with the 2008 World Health Organization classification.^14^ PDL1 testing was not performed. Responses were assessed with positron emission tomography (PET) scan and computed tomography (CT) scan every 3 months for the first year and then every 6 months for the second year of treatment. ORR (defined as the sum of complete and partial response rates at the end of treatment) was chosen as primary endpoint, whereas PFS and OS were analyzed as secondary endpoints. OS was defined as the time from initiation of therapy (CHT) to death from any cause and was censored at the date of last available follow up. PFS was measured from initiation of therapy (for both anti‐PD1 and subsequent CHT) to progression, relapse, or death from any cause.^15^ Responses were classified according to the Lugano criteria.^16^, ^17^ The toxicities were graded according to the National Cancer Institute Common Toxicity Criteria for Adverse Events (CTCAE version 4.0). No formal sample size estimation and power calculation were made for this observational retrospective study as we enrolled all patients treated at our Institute. Demographics and patients’ characteristics were summarized by descriptive statistics. Survival functions were estimated by using the Kaplan‐Meier method and were compared using log‐rank test. 95% confidence intervals (CI) were provided. Statistical analyses were performed with Stata 11 (StataCorp LP) and *P* values for statistical significance were set at .05.

## 3 RESULTS

### 3.1 Patients

Thirteen females and 12 males with a median age at diagnosis of 32.4 years (range 17.8‐67.1) were enrolled. According to Ann‐Arbor classification, 56% of patients (n = 14) had stage II and 44% (n = 11) had stage III/IV; B symptoms and bulky disease were counted in 14 and 7 patients, respectively. The study population was highly pretreated with a median of four prior therapies (1‐10), including ASCT (44%) and BV (92%). None of the 25 patients received allogeneic stem cell transplantation (alloSCT) before PD1 blockade. Twenty patients (80%) were refractory to the first line of treatment (for all patients ABVD: doxorubicin, bleomycin, vinblastine, dacarbazine). There was a high heterogeneity in the treatment given immediately before CPi, with the most common being BV as single agent (eight patients, 32%) or in combination with bendamustine (three patients, 12%), and ASCT (four patients, 16%). Twenty‐three patients (92%) were refractory to the last therapy before CPi (Table 1). At the start of anti‐PD1 therapy, the median age was 33.6 years (range 19.6‐72.0), 16 patients had Ann‐Arbor III/IV stage and 6 had B symptoms. Patients had anti‐PD1 therapy in the context of clinical trials (n = 21) or in the named patient program (n = 4): 15 out of 25 received pembrolizumab (3 patients at the dose of 10 mg/kg and 12 at the flat dose of 200 mg every 3 weeks) and the remaining underwent nivolumab (3 mg/kg every 2 weeks). A median of 14 cycles (range 3‐52) of anti‐PD1 therapy were infused (Table 1).

**TABLE 1:** Patient characteristics

Patient characteristics	N = 25
Sex, n (%)	
Male	12 (48)
Female	13 (52)
Median age at diagnosis, years (range)	32.4 (17.8‐67.1)
Histologic subtypes, n (%)	
Nodular sclerosis	19 (76)
Mixed cellularity	2 (8)
Lymphocyte rich	1(4)
Unknown	3 (12)
Ann Arbor stage at diagnosis, n (%)	
I	0 (0)
II	14 (56)
III	2 (8)
IV	9 (36)
B symptoms, n (%)	
Yes	14 (56)
No	11 (44)
Bulky disease, n (%)	
Yes	7 (28)
No	18 (72)
Prior therapies to PD‐1 inhibitor, median (range)	4 (1‐10)
ASCT, n (%)	11 (44)
BV, n (%)	23 (92)
Radiotherapy, n (%)	9 (36)
Allogeneic SCT, n (%)	0 (0)
Final response to the front‐line therapy (ABVD), n (%)	
Relapse	5 (20)
Refractory	20 (80)
Final response to the last therapy prior to PD‐1 inhibitor, n (%)	
Relapse	2 (8)
Refractory	23 (92)
PD‐1 inhibitor received, n (%)	
Pembrolizumab	15 (60)
Nivolumab	10 (40)
Number of cycles of PD1‐inhibitor, median (range)	14 (3‐52)
Best response to PD1‐inhibitor, n (%)	
CR	3 (12)
PR	16 (64)
SD	5 (20)
PD	1 (4)
Reason for CHT after anti‐PD‐1, n (%)	
PD	16 (64)
Sub‐optimal response (PR or SD)	7 (28)
Relapse	2 (8)

Abbreviations: ABVD, doxorubicin, bleomycin, vinblastine and dacarbazine; ASCT, autologous stem cell transplantation; BV, brentuximb vedotin; CHT, chemotherapy; CR, complete remission; PD, progressive disease; PR, partial remission; SD, stable disease.

Patient evaluation after start of CHT was done after a median of 3 months with PET and CT scan. The best response obtained with CPi was CR in 3 patients, PR in 16 patients, PD in 5 patients and stable disease (SD) in 1 patient. At the last PET and CT scan performed under treatment, 68% of patients did not respond to anti‐PD1 therapy (15 PD and 2 SD), whereas six had a PR and only two patients achieved a CR, with an ORR of 32%. Twenty‐three out of 25 patients discontinued the anti‐PD1 treatment due to or unsatisfactory response (21 patients, 15 in PD, 2 in SD and 4 in PR) or serious AEs (SAEs) (2 patients). In details, SAEs were a bronchiolitis obliterans with organizing pneumonia and an eosinophilic pneumonia, both resolved with steroids combined with CPi discontinuation.

### 3.2 Salvage therapy after PD‐1 blockade

After a median time of 1.5 months from the response assessment to anti‐PD1, 15 patients received a single agent CHT, whereas 10 had a multi‐agent treatment (Table 2). Overall, after a median of three cycles (range 1‐10), eight patients obtained a CR and seven a PR, with an ORR of 60%. Among the 15 patients who received a single agent CHT the ORR was 33% (1 CR and 4 PR), whereas, all the 10 patients treated with a multi‐agent regimen obtained a response with 7 CR and 3 PR (ORR 100%). In our series only three patients were re‐exposed to the same CHT agents that they have received before the CPi treatment, all of them were refractory at the first exposure and became responsive after anti‐PD1 therapy.

**TABLE 2:** Chemotherapy regimens after checkpoint inhibitor treatment

Pts	CHT before anti‐PD1	Response	Anti‐PD1	Response to anti‐PD1	First line CHT after anti‐PD1	N of cycles	Response	Re‐exposure to the same CHT	SCT	Response to SCT	Status at last follow‐up	Alive
1	BV	PD	Pembro	PD	BeGeV	4	CR	Benda; IGeV			CR	yes
2	BV + Benda	PD	Pembro	PD	BEACOPP	3	PR	Bleomycin, Adrymicin	ASCT	PR	CR	No
3	BV	PD	Pembro	PD	Benda	3	PD	No			SD	Yes
4	BV	PD	Nivo	PD	Benda	6	PD	No			CR	Yes
5	BV + Benda	PD	Nivo	PD	EDO101	3	PD	No			PD	No
6	IGeV	PD	Nivo	PR	PegDox	3	PD	No			SD	No
7	BV	PD	Nivo	PD	DHAP	3	PR	No	alloSCT	CR	CR	No
8	DHAP	PD	Nivo	PD	Daunorubicine	2	PR	No			CR	Yes
9	BEAM + ASCT	PD	Nivo	CR	PegDox	3	PD	No			PD	Yes
10	Melphalan + ASCT	PD	Nivo	CR	Benda	6	PR	No			CR	Yes
11	Lena + Benda	SD	Pembro	PD	PegDox	3	SD	No			CR	Yes
12	BV + Benda	PD	Pembro	PD	PegDox	5	PR	No	ASCT	CR	CR	Yes
13	DHAP	CR	Pembro	PD	EDO101	10	PR	No			PD	Yes
14	BV	PD	Pembro	PR	EDO101	5	SD	No			unk	Yes
15	Gemcitabine	PD	Pembro	PR	EDO101	6	SD	No			PD	Yes
16	Benda	CR	Pembro	PD	PegDox	4	CR	No	alloSCT	CR	CR	Yes
17	ABVD	PD	Pembro	PR	BeGeV	2	CR	No	ASCT	CR	CR	Yes
18	Vinblastine	PD	Nivo	PD	PegDox	2	PD	No			PD	No
19	DHAP	PR	Pembro	PD	ICE	2	PR	Ifosfamide	ASCT	PR	PD	No
20	BEAM + ASCT	PD	Nivo	PD	IGeV	3	CR	No	alloSCT	CR	CR	Yes
21	BV	PD	Pembro	PD	BEAM + ASCT	1	CR	No			CR	Yes
22	BV	PD	Pembro	PR	BEAM + ASCT	1	CR	No			CR	Yes
23	Radiotherapy	PD	Pembro	SD	PegDox	3	SD	No			unk	Yes
24	BV	PD	Pembro	PR	BEAM + ASCT	1	CR	No			CR	Yes
25	FEAM + ASCT	PD	Nivo	PD	BeGev	3	CR	No	alloSCT	CR	CR	Yes

Abbreviations: ABVD, adrimycin, belomycin, vinblastine, dacarbazine; alloSCT, allogenic stem cell transplantation; ASCT, autologous stem cell transplantation; BEACOPP, bleomycin, etoposide, adrimycin, cyclophosphamide, vincristine, procarbazine, prednisone; BEAM, carmustine, etoposide, cytarabine, melphalan; benda, bendamustine; CHT, chemotherapy; CR, complete response; DHAP, dexamethasone, high dose cytarabine, cisplatin; EDO‐101, first‐in‐class fusion molecule of an alkylator, bendamustine and the histone‐deacetylase inhibitor vorinostat; FEAM, fotemustine, etoposide, cytarabine, melphalan; ICE, ifosfamide, carboplatin, etoposide; IgeV, ifosfamide, gemcitabine, vinorelbine; Lena, lenalidomide; Nivo, nivolumab; PD, progression disease; PegDox, pegylated liposomal doxorubicin hydrochloride; Pembro, pembrolizumab; PR, partial response; Pts, patients; SCT, stem cell transplantation; SD, stable disease.

Sixteen out of 25 patients (64%) discontinued the salvage treatment: seven due to unsatisfactory response at the first evaluation (five PD and two SD), eight due to a consolidation with stem cell transplantation and only one patient due to a grade 3‐4 toxicity (febrile neutropenia with pneumonia).

Four patients (one in CR and three in PR) received ASCT and four had alloSCT (three patients were in CR and one in PR) as consolidation strategy. Among patients who underwent alloSCT: one patient received haploidentical transplant while the others received matched unrelated donor (MUD) transplantation with a reduced intensity conditioning regimen. The patient, who had haploidentical transplant, experienced a cutaneous grade 1‐2 acute graft vs. host disease (GVHD) treated and resolved with high‐dose steroids. Among the patients who received a MUD transplant, only one had a GVHD: she experienced cutaneous and intestinal grade 3 and liver grade 2 acute GVHD, resolved with high‐dose steroids and ruxolitinib. The transplant strategy allowed two patients to convert their PR into CR. Overall, 16 (64%) of the 25 patients who failed treatment with CPis achieved a CR, with a median of two lines of salvage CHT (range 1‐4). Seventeen patients (68%) experienced hematological toxicities: nine patients had neutropenia grade 3‐4, seven patients had thrombocytopenia (3 grade 1‐2 and 4 grade 3‐4, respectively), one patient had grade 1‐2 anemia. Six patients (24%) had extra‐hematological toxicities: two patients experienced grade 1‐2 fatigue, two patients had grade 1‐2 cutaneous rash, one patient had grade 1 diarrhea and one patient had a grade 3‐4 febrile neutropenia with pneumonia which resulted in treatment discontinuation.

### 3.3 Outcomes

**FIGURE 1:**
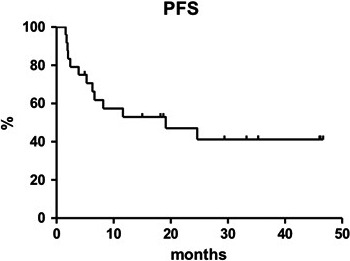
Progression‐free survival with chemotherapy post checkpoint inhibitor therapy

**FIGURE 2:**
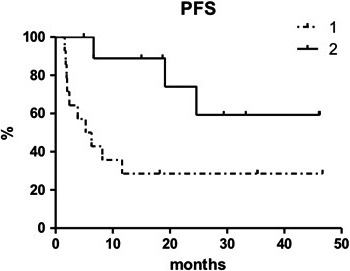
Progression‐free survival with salvage treatment (1: single agent; 2: multi‐agents regimen). Abbreviations: PFS, progression‐free survival

Median PFS with salvage treatment was reached at 19.1 months (Figure 1). PFS estimated from start of the salvage therapy was 41.2% at 3.8 years (95% CI 38.1‐44.3) and it was statistically higher in patients who underwent a multi‐agent regimen (59.3% [95% CI 57.2‐61.4] vs. 28.6% [95% CI 26.1‐31.1], respectively; *P* = .0252, Figure 2). After a median follow‐up of 32.4 months, 6 patients died (four due to a PD, one in CR due to secondary acute myeloid leukemia and the other also in CR due to pneumonia) and 13 patients are still alive and in CR. Eight out of these 13 patients are in continuous CR (CCR) after the first salvage treatment post PD1 blockade. The estimated OS from the start of the CHT was 56.5% at 3.8 years (median not reached, 95% CI 54.0‐59.0).

## 4 DISCUSSION

Immune CPis are transforming the standard of care across different tumor types leading to an improvement in the outcome and long‐term survival of a large subset of hematological patients. In particular, the inhibitors of PD1/PD‐L1 signaling has shown a good efficacy and a favorable toxicity profile in heavily pre‐treated HL and non‐Hodgkin lymphoma patients, with almost 70% of patients affected by Hodgkin disease reaching a response, including those who already underwent to ASCT and/or BV treatment.^7^, ^8^, ^9^, ^10^, ^11^, ^18^, ^19^ Despite these good results, a large part of the responder patients’ relapses, with a median PFS of maximum 12 months. Therefore, the attention is now focusing on how improving the response and on overcoming the resistance to CPis. Based on a rationale that DNA damaging agents are able to promote immunogenicity of cancer cells trough increasing neo‐antigen repertoire, inducing immunogenic cell death and changing the cytokine milieu into the tumor microenvironment, with a consequence redistribution and increase expression of PDL‐1 on tumor cells, good results are being achieved combining PD1 inhibitors with chemotherapy, both in solid tumors and in lymphomas setting.^20^, ^21^, ^22^, ^23^, ^24^ On the other side, patients who already failed anti‐PD1 therapy, seems to benefit from a re‐treatment with conventional CHT, leading to the idea that PD1 inhibitors can re‐sensitize tumor cells to conventional treatment, previously failed.^12^, ^13^ Rossi et al showed an overall response in 16 (67%) out of 30 R/R HL patients treated with conventional CHT after anti‐PD1 treatment (CR: 46%), regardless of whether patients had been re‐exposed to an agent to which they were previously resistant. A trend to a better response was seen in those patients in which CHT was combined to anti‐PD1 in comparison to those in which CHT was administered after stopping PD1 inhibitors, underlining again a potential synergy between these two approaches.^12^ In a retrospective analysis from seventeen centers across US and Canada, 77 R/R HL patients received different type of salvage treatment after CPi failure. The ORR was 52% (17 CR and 9 PR) with a median PFS of 10.7 months. The authors pointed out that a response to salvage treatment appears to correlate with response to CPi itself, although a PD following anti‐PD1 therapy did not preclude a response to subsequent CHT.^13^ In our analysis, all the 25 R/R HL patients underwent conventional CHT only after quitting anti‐PD1 treatment and 15 patients (60%) achieved a response (eight CR and seven PR) to the first salvage treatment. Among the responder patients, eight underwent SCT, consolidating their response. We observed a better improvement of ORR (100% vs 33%) and a statistically higher PFS at 3.4 years (59.3% vs. 28.6%, respectively; *P* = .0252) in patients treated with a multi‐agent regimen compared to those treated with single agent. No differences in term of response to salvage treatment were seen between those who relapsed after or were refractory to CPi. After a median follow‐up of 32.4 months, 13 patients are still alive and in CR, with 8 patients still in CCR after first salvage treatment post CPi. To note, in this heavily pre‐treated population the median OS was not reached.

Our results are in line with what previously observed, supporting the hypothesis of a new chemo‐sensitization due to anti‐PD1 treatment in HL patients with highly pre‐treated and chemo‐refractory disease. This approach gave also a chance for some patients to receive consolidation with SCT (both allogeneic and autologous), increasing the likelihood of being cured. Further studies are needed to better understand the biological rationale of the new chemo‐sensitization after CPi and to establish the most appropriate chemotherapy regimen and the best timing of CHT and anti‐PD1 administration in the R/R cHL therapeutic algorithm.

## CONFLICT OF INTEREST

Authors declare no conflict of interest.

## AUTHOR CONTRIBUTIONS

BC, LA, AM, MC, and PLZ contributed to conception and design. LA contributed to data Analysis. BC, LA, and PLZ contributed to interpretation of the data. BC, LA, and PLZ contributed to drafting of the article. BC, LA, MC, and PLZ contributed to critical revision for important intellectual content. BC, AM, GL, AB, CP, LN, VS, MC, and PLZ contributed to provision of study materials or patients. BC, LA, AM, GL, AB, CP, LN, VS, PEC, and M.C contributed to collection and assembly of data. All authors participated in writing and/or critically reviewing the manuscript and approved this version for submission.

## Data Availability

The datasets used and analyzed during the current study are available from the corresponding authors on reasonable request.
